# Training Early Childhood Teachers to Support Children’s Social and Emotional Learning: A Preliminary Evaluation of Roundies Program

**DOI:** 10.3390/ijerph182010679

**Published:** 2021-10-12

**Authors:** Ali Moazami-Goodarzi, Maryam Zarra-Nezhad, Maija Hytti, Nina Heiskanen, Nina Sajaniemi

**Affiliations:** 1 School of Applied Education Science and Teacher Education, University of Eastern Finland, 80101 Joensuu, Finland; ali.moazami@uef.fi (A.M.-G.); nina.heiskanen@uef.fi (N.H.); nina.sajaniemi@uef.fi (N.S.); 2 Rounders Entertainment Oy, 00120 Helsinki, Finland; maija.hytti@roundies.fi

**Keywords:** pilot study, intervention program, social-emotional learning, early childhood education and care, professional development

## Abstract

(1) Background: Implementing social-emotional learning (SEL) programs in Early Childhood Education (ECE) settings is a promising approach that can strengthen overall development and well-being during childhood and into adolescence and adulthood. This study described the development, implementation, and preliminary evaluation of a universal SEL program, i.e., Roundies, in the Finnish ECE context to address the need for professional development opportunities. (2) Methods: The Roundies program was a quasi-experimental pre-test/post-test with intervention and control design. A total of 194 children were assigned to either the intervention group (*n* = 136) or control group (*n* = 58) (M_AGE_ = 60.35 months at pre-test; 48% boys). Teachers rated the children’s behaviors using the Strength and Difficulties Questionnaire (SDQ) at the pre-and post-test. (3) Results: Feedback collected by teachers on the program suggested that teachers were highly satisfied with the overall program and the components. Multilevel models showed significantly increased prosocial behaviors and reduced SDQ total difficulties in the intervention group compared to the control group. (4) Conclusions: These preliminary findings provide evidence of the effectiveness of the Roundies program in improving teachers’ capacity to support early SEL.

## 1. Introduction

Early social-emotional learning (SEL) refers to a broad range of social, emotional, and behavioral skills. SEL is critical to children’s adaptive development, well-being, positive peer relations, school readiness, and academic success [1], in addition to adjustment and mental health into adolescence and adulthood [2]. In Europe, 95% of children aged four and over spend most of their weekdays (on average, 29.5 h) in Early Childhood Education (ECE) systems [3]. Therefore, early childhood professionals’ knowledge, skills, and practices are crucial factors affecting children’s learning and development during the first years of life and beyond [4]. Early intervention programs in ECE centers aimed at improving professional development (PD) to promote children’s social-emotional competencies thus deserve more attention.

According to a Finnish national survey on ECE’s social-emotional competencies [5], of 94 teachers, 11% reported increased social-emotional challenges and restlessness in early childhood. Further, 12.6% (from 6910 children) of the 3–6-year-olds in Finnish ECE centers were reported (by parents, teachers, and child interviews) as being involved in bullying [6]. However, PD opportunities to ensure teachers have the essential knowledge and skills to promote early childhood social-emotional competence are limited [7,8,9,10,11]. In Finland, the increasing need to enhance ECE teachers’ professional competence through further training has been widely recognized [12]. Further, there is a pressing need for reporting the effectiveness of early PD interventions in Europe, particularly in Nordic countries [5,11]. Consequently, this pilot study aimed to describe the development, implementation, and primary evaluation of a universal curriculum-based SEL intervention program, i.e., Roundies, in the Finnish ECE context to address these gaps. This program has been developed for the first time in Finland to serve the growing need for pedagogical programs to increase teachers’ understanding of early social-emotional development and provide them with the materials and practical curriculum guide for supporting children’s early SEL.

### 1.1. Promoting SEL in Early Childhood

The Collaborative for Academic, Social, and Emotional Learning (CASEL) defined SEL as the process through which children acquire and apply the knowledge, skills, and attitudes needed to establish and maintain positive interactions with others, understand and manage their own emotions, recognize other’s emotions, feel and show empathy for others, set and achieve positive goals, and make responsible choices [1,13]. According to CASEL, an effective SEL program aims to foster the early development of a core set of five competencies that provide a foundation for optimal growth. These include: (1) Self-awareness, i.e., recognizing and naming own emotions, understanding own needs, and being aware of one’s own thinking and behavior. (2) Self-regulation, i.e., the ability to regulate one’s own emotions, thoughts, and behaviors in different situations, through remembering and generalizing what they have been taught, initiating changes in their behavior, and continuously monitoring their behavior, so that goals are achieved. These skills are emerging during the preschool years as the brain develops. (3) Social awareness, i.e., the ability to recognize and understand others’ feelings and emotions, empathize with them, and see things from their viewpoints. (4) Relationship skills, i.e., the ability to establish and maintain positive relationships with peers and adults, effectively engage in social communication, cooperate with others, listen attentively, and seek and offer help when needed. (5) Responsible decision making, i.e., the emerging ability to make positive choices about personal and social behavior.

The core social-emotional competencies provide children with the foundation needed to regulate their emotions, build secure and stable social relationships, cope with challenges, solve problems, and successfully adjust to early education settings [2,14]. However, it is essential to remember that young children are still fragile and need scaffolding to regulate emotion in complex social environments. Lack of adaptive social-emotional competencies, conversely, may put children at increased risk of developing severe forms of behavioral problems, including internalizing problems (i.e., where negative emotions are directed at oneself) such as the feeling of loneliness, depression, and anxiety [15], and externalizing problems (i.e., when negative emotions and social actions are directed against others) such as antisocial behavior, aggressiveness, conduct problems, and bullying [15]. Jones et al. [2] found that early childhood social-emotional competence is linked to mental health, academic outcomes, employment, criminal activity, and substance abuse 13 to 19 years later. They suggested that children who have stronger social-emotional competencies are more likely to become well-adjusted adults. Therefore, SEL interventions can significantly affect children’s development [16].

### 1.2. Supporting ECE Teachers

ECE educators play a crucial role in facilitating SEL skills [9,17], given the developmental age of the children, who are undergoing a rapid increase in social, emotional, and cognitive development [18,19]. Teachers are the engine that drives SEL programs, and teacher–child interaction plays a critical role in creating a supportive and equitable learning environment [17]. According to the bioecological model of human development [20], development is an ongoing interactive process between the child’s characteristics and the close context—the so-called proximal processes of development. Considering this model in an educational setting, teacher–child interaction is the primary proximal process that can positively or negatively affect a child’s development [21]. Thus, child behavior and SEL skills are fundamentally influenced by the quality of teacher–child interaction and teachers’ behavior. The extent to which a teacher can adjust the learning opportunities to an individual child and their specific characteristics is a critical factor for promoting the child’s academic, cognitive, and social development [21].

High-quality teacher–child interactions are positively related to essential child outcomes such as growth in learning competencies [22], social-emotional skills [23], and self-regulation [24]. However, fostering and maintaining supportive child–teacher relationships is challenging, and teachers need adequate SEL-promotive skills, competencies, and strategies when engaging with the children [19]. In fact, addressing young children with compromised social and emotional skills is one of the challenges most often expressed by early childhood teachers [9]. Therefore, there is growing interest in implementing SEL programs to influence child outcomes by strengthening teachers’ capacity and capability to implement SEL practices with fidelity [25].

The Finnish ECE Curriculum [26] includes various aspects and purposes of learning social-emotional skills and the importance of supporting SEL. However, SEL skills are not clearly separated as their own area of learning. Therefore, it is essential to introduce effective SEL programs in early childhood. The Finnish Center for Educational Evaluation (FINEEC) has studied ECE’s quality and the implementation of ECE curricula in Finland [27]. The findings revealed that the curriculum’s implementation is influenced by both leadership structures and sufficiently concrete pedagogical practices. The pedagogical knowledge and continuing education of the staff are also identified as critical elements in this process. Therefore, pedagogical practices programs must be justified and adequate, their implementation examined, and the curriculums further developed to meet the learning needs of the future. Implementing an effective SEL curriculum can effectively develop teachers’ attitudes, mindsets, and perceptions about early SEL. It also can equip them with sufficient resources and practices to positively evaluate their abilities to handle classroom situations, perceive children’s behavior as less challenging, and improve children’s SEL [7]. A recent systematic review suggests that universal curriculum-based SEL programs in ECE settings may strengthen teaching practice and behavior, particularly effective classroom management and responsive and nurturing teacher–child interactions [25]. Despite growing recognition of the importance of young children’s social-emotional development, fewer PD opportunities or studies supporting early childhood teachers to serve that role have been reported in Europe [11]. For example, two studies implemented the VIDA intervention over about 1½–2 years on 3–6-year-old children’s social-emotional behaviors (measured by SDQ) in Denmark [28,29]. The results revealed that the intervention had a positive effect on children’s behavior due to the PD of the preschool educators. Another study by Perels, Merget-Kullmann, Wende, Schmitz, and Buchbinder [30] examined the impact of a 5 week PD intervention on 5–6 years old children’s self-regulation in Germany. The results indicated that the training program for teachers improved the self-regulated learning of preschool children.

### 1.3. The Roundies Intervention Program

The Roundies program, Learning Social-Emotional Skills the Fun Way, is a new cross-media concept focusing on soft skills during early childhood. It has been developed in Finland to enhance social-emotional competencies and prevent early-onset behavioral problems in 3–6 year old children (Korkeamäki, Hytti, Pöyhönen, & Livingston, 2017–2019; http://tunteetjataidot.fi, accessed on 16 September 2021). It has been developed in conjunction with pedagogical and psychological experts and was tested in the Playful Learning Center of the University of Helsinki. The concept of the Roundies program is derived from positive psychology and SEL principles. The program’s curriculum is based on the CASEL framework and in line with Finnish law and the national core curriculum for early childhood education and care (ECEC) [31]. The Finnish ECE includes ten goals and is guided by the Act on ECEC [31]. It is based on a holistic approach, i.e., “EDUCARE”—care, education, and teaching, where a child’s development and learning are seen holistically. The eighth goal in the law refers to SEL, having the aim that ECEC needs to develop children’s cooperation and interaction skills and promote ethical and responsible behavior. Consequently, the Roundies program was designed to support children’s SEL and development, and promote a positive classroom environment where learning is seen as playful, insightful, and participatory. A universal approach was chosen targeting all children, independent of their risk status, which avoids stigma, reduces or prevents various behavior problems, and promotes a broad range of protective factors [32].

The SEL intervention core components in Roundies are designed to reinforce children’s social-emotional competency focusing on: (1) promoting the pedagogical sensitivity of ECE professionals; (2) promoting children’s social-emotional competencies such as self-regulation skills, prosocial skills, interpersonal relationships, stress-tolerance, and positive thinking; and (3) reducing behavioral problems in early childhood in terms of internalizing and externalizing problems, emotional distress, and bullying. The core elements of the Roundies program consist of eight sessions (55 min each) for ECE professionals (in this case, teachers) and 19 sessions (45 min each) for children. Teacher sessions include an educational portal with training videos and downloaded materials on promoting children’s socio-emotional competencies and dealing with children’s challenging behaviors in everyday life situations. Teachers are provided with the appropriate materials, techniques, and practical curriculum guide to support SEL in the classrooms. Children sessions include SEL learning activities such as singing and breathing exercises and learning materials such as Roundies storybooks and emotion cards. There are six Roundies storybooks in the program written around the core themes of SEL. The animal characters of the books have their own characteristics and emotions that children can relate to. Using the stories as a basis for different SEL activities, discussion, and educational drama methods creates a rich environment promoting children’s social-emotional skills. The Roundies program was designed to be effective by dividing the sessions into smaller themes and providing enough time and variety on each theme, including active learning methods, reflection, feedback, and meta-discussions with the children about the sessions’ goals. This structure falls into the guidelines of the CASEL framework [1].

### 1.4. The Present Study

This pilot study aimed to add knowledge in the field of ECEC by addressing PD opportunities that enhance early childhood teachers’ knowledge and skills to effectively apply SEL in the classrooms. This study describes the development of the Roundies program, the initial implementation, its preliminary evaluation results, and the feasibility and usefulness of the program to increase the probability of success in the full-scale study. A quasi-experimental design was conducted with an intervention group (IG) and control group (CG) that provided data at a pre-test and post-test. The study posed the following research questions: (1) whether teachers are satisfied with the program; (2) whether the teacher’s PD promotes children’s prosocial behaviors in ECE environments; and (3) whether the teacher’s PD decreases children’s behavioral problems.

## 2. Materials and Methods

### 2.1. Participants and Procedure

**Figure 1:**
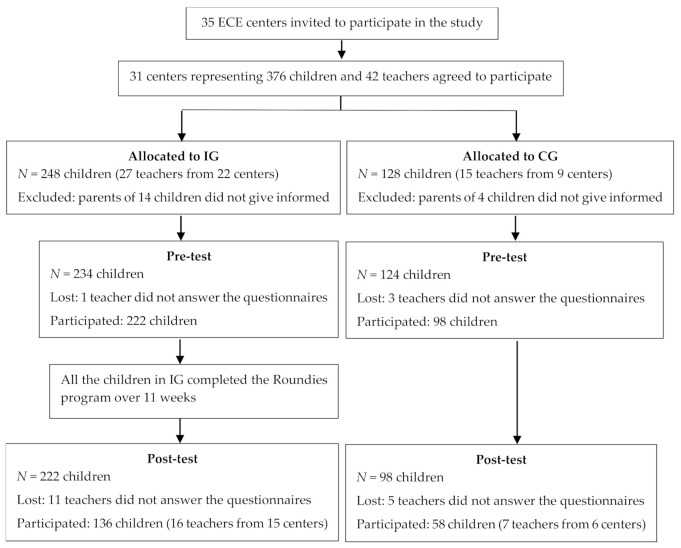
Flowchart of the study design and the number of corresponding participants.

The present study was carried out in ECE centers in Finland during 2019–2020. The sample size was initially calculated to detect emotional and behavioral changes in 3–6 year old children for power of 0.8, a two-tailed α of 0.05, and an effect size of 0.5. The power analysis indicated that 49 children were needed in each group. To allow for dropouts, the target size was 70 children in each group. ECE centers from seven municipalities were invited by the research party to participate in the study. A total of 31 ECE centers agreed and consented to participate in the current study (the ECE centers participated with one to three groups each). Then, these ECE centers received more information about the study. From 376 eligible children, those who were not available for further assessment were excluded. Overall, 95% of the parents provided their written informed consent for their children’s participation in the study. Consequently, final data were available for 320 children at the pre-test (before program implementation) and 194 at the post-test (after full program implementation). A flowchart of the study design and the number of participants is presented in Figure 1.

The mean number of children in the household was 2.64 (SD = 0.99), and the child’s mean age was 61.69 months (SD = 12.26). The majority of children in the sample (76%) came from nuclear families, and 22% were from single-parent families. At the baseline, 80% of parents were employed, and 15% were housewives/househusbands. A total of 60% of parents had a university degree, and 40% had secondary education. The sample was reasonably representative of the level of education among the general population in Finland [33].

The program was evaluated using an 11 week trial design with the IG and CG. After stratified selection based on relevant socio-demographic variables, recruitment, and random assignment to the IG and CG, 136 children (from 16 ECE centers) belonged to the IG, and 58 children (from 7 ECE centers) belonged to the CG during the evaluation period. A non-significant chi-square test for gender (49% girls in the CG vs. 62% girls in the IG; *p* = 0.061) and a non-significant *t*-test and small effect size for age variable (58.52 months in the CG vs. 60.95 months in the IG; *p* = 0.20) supported the comparability of the intervention and control group. Teachers in IG were trained in the implementation of the program. Teachers in the CG did not receive any training and continued with their normal pedagogical activities in their ECE center. Children’s data were collected by teachers’ reports, using similar measures at pre- and post-test. In the IG, data were collected before the teacher-training phase (pre-test) and after the program’s full implementation (post-test). Independent *t*-test results for the outcome variables showed that the intervention group and control group differed significantly on the total SDQ difficulties at T1 (*t* (192) = 2.62, *p* = 0.009). No differences were found for the prosocial at T1 between two groups (*t* (192) = 1.33, *p* = 0.185). Hence, the pre-test score for outcome variables was used as covariates in multilevel model analysis. A detailed comparison between the IG and CG is presented in Table 1.

Before the program implementation, the plans, questionnaires, information letters to parents, and consent forms were circulated. Informed consent was sent to the children’s parent(s)/guardian(s) before the intervention. In this study, all parts were carefully carried out following the ethical principles of the Helsinki Declaration and the Finnish Advisory Board on Research Integrity. An informed consent form was sent to the heads of the ECE centers who were approached to participate in the study and later to the children’s parent(s)/guardian(s) to sign before the intervention. The participants were recruited voluntarily and guaranteed full anonymity, and could withdraw themselves and all their information from the study without reason. The Roundies SEL curriculum supports the content of the ECE plan and can be implemented as part of the normal pedagogical activities of every ECE center. Therefore, the benefits of the intervention are shared by all children on an equal basis. The decision of the parents/guardians to participate in the study does not affect whether children or parents/guardians receive the activity or materials for the intervention. Measurements included in the effectiveness of the intervention were only collected from consenting children. ECE staff participated in the intervention study during their working hours as part of their work, and it did not involve any additional work.

### 2.2. Measures

Teachers’ satisfaction with the program. At the post-test, teachers answered six questions about the helpfulness of the program in general. They were asked to rate the impact of the program on their classroom (e.g., The program made some changes that improved the way things work in my classroom; I can see improvement in children’s emotional expressions in the classroom) on a 5 point rating scale (1 = Strongly disagree, 5 = Strongly agree). Teachers were also asked to rate the helpfulness of each program component (i.e., the handbook, online learning materials, teacher discussion groups, toolkit items designed to help teacher practice) on a 2 point rating scale (1 = not helpful, 2 = helpful).

The Strength and Difficulties Questionnaire. Teachers and parents completed the Finnish version of the Strength and Difficulties Questionnaire (SDQ) at the pre-and post-test. The SDQ consists of 25 items and five subscales, each containing five items: emotional problems (e.g., “many fears and easily scared”), conduct problems (e.g., “often fights with other children or bullies them”), hyperactivity/inattention (e.g., “restless, overactive, and cannot stay still for long”), peer relationship problems (e.g., “rather solitary and tends to play alone”), and prosocial behaviors (e.g.,” shares readily with other children”). Teachers were asked to rate each child on a 3 point rating scale (1 = not true, 2 = somewhat true, 3 = certainly true). Subscales were created by calculating the sum score of relevant items. The SDQ total difficulties score was computed by summing the four problem behavior subscales (emotional problems, conduct problems, hyperactivity, and peer relationship problems). The Cronbach’s values of all subscales in both samples at both the pre-test and the post-test ranged from 0.72 to 0.86.

### 2.3. Impementation of the Roundies Intervention Program

The program consists of eight sessions (55 min each) for teachers and 19 sessions (45 min each) for children. The sessions are divided into six themes to cover the core set of five SEL competencies, i.e., self-awareness, self-regulation, social awareness, relationship skills, and responsible decision making. Each theme begins with a session for teachers, followed by three sessions for children (see Table 2). Teachers were asked to complete each theme within two weeks.

The program started with a teacher-orientation session, introducing the Roundies program goals and the implementation of the SEL practices in the classroom. Next, each of the six themes began with one teacher session. For each teacher session, the teachers educated themselves on the topic using online learning materials, discussions, and the handbook. Then teachers prepared the three children’s sessions for each theme, according to the handbook instructions. The learning materials for children’s sessions included six printed children’s storybooks, strengths cards, emotion cards, breathing cards, different posters, and diplomas. These sessions consisted of discussions, drama education, singing, physical exercises, art assignments, and breathing exercises. Each children’s session also included one “Emotion of the day”, which focused on emotions such as joy, pride, happiness, anger, guilt, and sadness. After each theme session, the teachers were asked to evaluate the theme online and give feedback. The children’s final session was named the “End celebration,” where they performed some fun exercises and received diplomas for participating in the intervention program.

### 2.4. Professional Development Training of Teachers

The Roundies program was developed because of the need for a pedagogical program that provides teachers (and parents) with well-designed material, practical guidance, and theoretical knowledge to enhance SEL, reduce existing behavioral problems, and prevent social-emotional skill deficits in early childhood. It uses easy-to-apply elements that can be integrated into everyday practices. The teachers involved in the program were trained in implementing the program through eight workshop sessions by online videos over the Roundies online portal and the program’s handbook. The handbook includes theoretical information, instructions, exercises, and some tips on applying theoretical knowledge in the classroom. Teachers also had regular face-to-face meetings, including “update days”, to maintain the training quality and implementation fidelity. The detailed descriptions of the eight teacher training sessions that address the five components of SEL are presented in Table 3, Supplementary Material File S1.

### 2.5. Statistical Analysis Approach

This study was considered to be a multilevel design due to the nesting of the unit of analysis (observations) within the experimental unit (classroom teacher). In datasets with a hierarchical structure, individuals are not entirely independent because respondents in the same unit tend to be similar. This assumption strongly determines the nature of the statistical analysis procedures to be adopted when studying intervention effects. According to Hox [34], ignoring the hierarchical structure of the data leads to underestimating standard error and many spuriously significant results. Multilevel modeling accounted for nesting effects within the statistical procedures and decreased the risk for Type-1 errors, providing a more rigorous statistical technique where multilevel designs occur [35,36]. To examine the nesting effect, an intra-class correlation coefficient (ICC) measured the amount of variance in within-level post-test scores attributed to the between level. If the ICC is below 5%, then nesting effects are not present and multilevel procedures are not necessary [37].

In the present study, a total of 194 children (136 in IG and 58 in CG), were nested within 23 teachers (M_AGE_ = 39.05 years, SD = 8.39 at pre-test; 100% female). At level 1, variables relating to the children, i.e., pre-test score, gender, and age, were considered. At level 2, the experimental condition variable was included, and CG was regarded as the reference group. All the statistical analyses (including descriptive and multilevel modeling) were performed via SPSS statistics (version 26) software and the models estimated with maximum likelihood. The dependent variables yielded desirable univariate *z* statistics for skewness (*z* between −2.58 and +2.58) indicating that variables were normally distributed. Based on a step-by-step procedure suggested by Hox [34], four multilevel models were built and compared. Model 1 is the intercept-only model (or unconditional model) with no explanatory variables. It shows how the total variance of the post-test score is split into between-level variance (differences between classroom teachers) and within-level variance (differences between timepoints). Model 2 includes all lower-level explanatory variables as fixed and with non-random slopes. In Model 3, the level 2 factors were added. This model examines whether the level 1 explanatory variables explained between-level variation in the post-test score. Finally, in Model 4, the interaction term of the condition by pre-test was added to determine whether significant difference conditions depend on pre-test scores. In all models, the intercept refers to the expected mean post-test score when all predictors are zero. Model improvement is assessed by a significant decrease in the deviance value compared to the previous model. All results are reported on the basis of intention to treat.

## 3. Results

Teachers’ feedback revealed that 97% of teachers were satisfied with the overall program. Teachers reported improved teacher–child relationships, and positive social and emotional behaviors in children. They also reported that they will continue using the program in the future and would recommend it to other teachers. The program’s toolkit items (e.g., the emotional cards, structured discussions, the Roundies storybooks) were the most positively rated program components from the teachers’ perspective (with more than 95%). Table 4 shows teachers’ satisfaction with the Roundies program in general and their satisfaction with the individual components. Further, to determine the overall impact of the Roundies program on children’s social-emotional behaviors, represented by the five SDQ subscales, multilevel modeling was applied.

### 3.1. Prosocial Behaviors

Model 1 indicated that the between-level variance explains 40% (0.47/0.47 + 0.72) of the variance in prosocial scores at post-test, which is sufficient to use multilevel modeling. Models 2 to 4 showed how the intraclass and interclass variance proportions varied when explanatory variables were introduced. First, the shift from Model 1 to Model 2 led to the largest reductions in variance proportions, which explained 46% [(0.72 − 0.40)/0.72] of the within-level variance of post-test scores and 54% [(0.47 − 0.22)/0.47] of the between-level variance of post-test scores. In this model, the pre-test score variable was statistically significant, suggesting that post-test scores were highly dependent on the initial prosocial score. More precisely, when all other level 1 variables were kept constant, each increase of one unit of the pre-test score was associated with an increase of 0.71 in the post-test score. Further, gender and age variables were statistically significant at *p* < 0.05 (b = −31, b = 0.01, respectively). Taking children’s pre-test scores into account, boys showed less prosocial behaviors than girls on post-test. Model 3 did not explain more within-level however, it explained 71% of the between-level variance in prosocial scores at post-test. The effect of the experimental condition was statistically significant (b = 0.65, *p* = 0.037) such that the prosocial scores at post-test were higher for the IG than the CG. In Model 4, which explained 32% [(0.72 − 0.41)/0.72] of the within-level variance of post-test scores and 71% [(0.47 − 0.14)/0.478] of the between-level variance of post-test scores, the interaction terms between the pre-test score and the condition were added. The interaction was not statistically significant at *p* < 0.05 (b = −0.037), meaning that the prosocial behaviors scores were higher at post-test for the IG. This suggests that the effect did not differ according to the initial level of prosocial behaviors (see Table 5).

### 3.2. The SDQ Total Difficulties

Model 1 indicated that the between-level variance explains 39% (0.31/0.31 + 0.492) of variance in SDQ total difficulties behaviors at post-test, which is sufficient to use multilevel modeling. Model 2 led to the most significant reductions in variance proportions, which explained 64% [(0.49 − 0.17)/0.49] of the within-level variance of post-test scores and 81% [(0.31 − 0.03)/0.31] of the between-level variance of post-test scores. In this model, the pre-test score variable was statistically significant, suggesting that post-test scores were highly dependent on initial SDQ total difficulties; that is, when all other level 1 variables were kept constant, each increase of one unit of the pre-test score was associated with an increase of 0.816 in the post-test score. Model 3 did not explain more within-level variance; however, it explained 89% of the between-level variance in SDQ total difficulties scores at post-test. The effect of the experimental condition was statistically significant (b = −0.34, *p* = 0.033) such that the SDQ total difficulties score at post-test was lower for the IG than the CG. In Model 4, which explained 64% [(0.49 − 0.17)/0.49] of the within-level variance of post-test scores and 90% [(0.31 − 0.03)/0.31] of the between-level variance of post-test scores, the interaction terms between the pre-test score and the condition were added. The main effect of the intervention was still negative and significant at *p* < 0.05, but the interaction was not. This indicates that the SDQ total difficulties score was lower at post-test for IG. In addition, the non-significant interaction term suggests that the effect did not differ according to the initial level of SDQ total difficulties (see Table 6).

## 4. Discussion

This study shared a PD approach supporting children’s social and emotional development, consistent with the CASEL framework based on the theory and developmental research on social and emotional competencies. The goal was to describe the Roundies SEL program’s development and implementation, and report the preliminary evaluation results on children’s social-emotional behaviors.

Based on the evidence, teachers were highly satisfied with the Roundies program and found the program components, particularly the toolkit items (e.g., emotional cards, structured discussions, Flamingo’s nest, and the Roundies storybooks) very helpful for children’s SEL. Our results further revealed that children who received the Roundies program (IG) showed a more significant increase in their prosocial behaviors (e.g., helping, sharing, and cooperating) over time compared to the CG. Furthermore, the results showed that for children in the IG, a more significant decrease in SDQ total difficulties over time was evident compared to the CG. This indicates a promising application of the program for promoting early childhood SEL. In line with Finnish law and the national core curriculum for ECEC [31], the Roundies SEL program aimed at developing children’s interpersonal and interaction abilities, promoting their relationship skills to function positively in peer groups, and guiding them towards ethically responsible and sustainable actions, respect other people, and membership in society (aim 8).

Our findings are consistent with ecological and transactional models of development [20], suggesting that children’s interactions with teachers and peers in the classroom context are considered proximal influences on their social and behavioral development [38]. Teachers’ PD plays a critical role in promoting young children’s social and emotional competence [7,8,9,11,39]. Therefore, PD requires research-based information about appropriate practices and adequate support for developing teachers’ reflection and understanding of early SEL and development. With systematic professional learning, teachers learn to observe the variation of children’s social-emotional competence more broadly from a socio-cultural perspective and based on children’s needs [9]. Therefore, the Roundies program has equipped teachers with adequate SEL-promotive skills, competencies, and strategies to implement effective classroom management techniques and responsive and nurturing teacher–child interactions. This then helped teachers create a positive learning environment, leading to stronger prosocial skills and fewer behavioral problems in children. Our findings further highlight the fundamental role of teachers’ relationships with children as a critical predictor of children’s later social-emotional competence and academic achievement. It may be that the Roundies program improves dispositional mindfulness among teachers. Increasing dispositional mindfulness has been linked with higher-quality relationships, less stress, and decreased workplace conflicts in early childhood education environments [40]. Quality relationships can be defined as a teacher’s ability to create an emotionally supportive environment by using a warm tone and positive affect in communication, considering children’s perspectives, giving them autonomy, and being sensitive to their needs and emotions [41], which are the aims of the Roundies program.

Interestingly, our results showed that 40 percent of the variance in prosocial scores resulted from differences among teachers. We assume that differences in teachers’ social-emotional competence at baseline or their fidelity to the program may explain the variation. Future research is needed to take the teacher-level and preschool-level characteristics into account.

Although both girls and boys showed improved social-emotional skills after the intervention, our results suggested that girls may benefit more than boys in terms of prosocial behaviors. Girls tend to show less of an out-group bias, as they behave more generously toward a least-liked peer than do boys [42]. It has been found that girls tend to give more, which may stem from societal norms and socialization practices that emphasize generosity and caretaking for girls [43]. In addition, there are differences in the development of self-regulation between boys and girls [44]. Further, boys are more prone to receive a diagnosis of ADHD than girls [45]. It seems that ECE environments, in general, boost socio-emotional development in girls more than among boys. Hence, it is vital to acknowledge the need to promote gender awareness in ECE professionals and support them in being open to boys’ and girls’ choices in learning and development and to engage boys in SEL activities if they seem uninterested compared to girls. This raises the need to promote gender equality within and through education, and provide girl-friendly and boy-friendly pedagogies.

The present study has some limitations that are worth mentioning. First, children’s behavioral outcomes have been assessed using only teachers’ reports. Although teacher ratings are reliable indicators of social-emotional behaviors, future studies may include age-appropriate self-reports, direct behavioral observations, or parental ratings of social competence to yield stronger results. Second, this study is based on a non-random, small sample; the sample size, particularly for the CG, was not large enough to extrapolate the results to the general population. Moreover, the small sample limited the validity of this pilot study. Full-scale intervention studies would benefit from larger-scale studies employing random sampling procedures, and power analysis can ensure the required sample size. Third, attrition bias regarding missing outcome data was considerably high due to a large number of dropouts. This high number of dropouts was mainly due to the COVID-19 situation or changes in ECE centers’ personnel. Some teachers either tested positive themselves or had to self-isolate due to being around someone who tested positive and therefore had to leave the intervention. Further, some teachers had changed either the child group they were working with or left the center piloting the Roundies program and therefore did not participate in the post-test. However, in this pilot study, we used only the paired (pre- and post-measurement) data files, which may be influenced by selection bias. Finally, an obvious gender bias must be considered because all the participant teachers were female. Therefore, the results of this study should be interpreted with caution.

## 5. Conclusions

The results of this pilot study suggest that the Roundies program may be a promising approach in providing PD opportunities that enhance early childhood teachers’ knowledge and skill in effectively applying SEL in the classrooms. Teachers were highly satisfied with the Roundies program and found the program components, particularly the toolkit items, to be very helpful. The multilevel regression analyses revealed that children in the IG showed a more significant increase in prosocial behaviors and a more significant decrease in the SDQ total difficulties over time compared to the CG. This indicates a promising application of the program for promoting early childhood SEL. Our results also suggest that girls may benefit more from the program in terms of prosocial behaviors compared to boys. This raises the need to promote gender sensitivity and equality within and through education by providing girl-friendly and boy-friendly pedagogies in ECE settings.

## Figures and Tables

**Table 1:** Descriptive statistics and *t*-test results of SDQ.

	CG (*n* = 58)		IG (*n* = 136)		*t*	*df*	*Sig.*	*d*
	Mean	SD	Mean	SD				
Age	58.52	14.76	60.95	11.56	−0.91	115	0.361	0.20
Prosocial T1	4.16	0.94	4.55	1.02	−2.50	192	0.013	0.39
Prosocial T2	4.11	0.98	4.83	1.10	−4.30			
Emotional problems T1	2.74	1.27	2.50	1.10	1.33	192	0.185	0.21
Emotional problems T2	2.59	1.14	2.42	1.09	0.99			
Conduct problems T1	2.85	1.09	2.56	1.11	1.70	192	0.090	0.27
Conduct problems T2	2.79	1.15	2.44	1.15	1.94			
Hyperactivity T1	3.92	1.37	3.43	1.40	2.21	192	0.028	0.35
Hyperactivity T2	4.07	1.48	3.25	1.34	3.79			
Peer problems T1	3.18	1.09	2.81	1.08	2.22	192	0.028	0.35
Peer problems T2	3.31	1.06	2.68	1.11	3.63			
Total SDQ difficulties T1	3.17	0.78	2.82	0.87	2.62	192	0.009	0.41
Total SDQ difficulties T2	3.19	0.78	2.70	0.91	3.59			
Gender (boys %)	62%		49%		*(x*^2^ = 3.505)	1	0.061	

**Table 2:** The Roundies curriculum themes and description of teachers’ and children’s sessions.

Program Themes	Teachers’ Session	Session 1 for Children	Session 2 for Children	Session 3 for Children
Self-awareness	Becoming familiar with: − Self-awareness & strengths − Child’s temperament − Separation anxiety	The storybook: “Three Cheers for Giraffe” promotes encouragement, overcoming fears, and finding strengths. The emotion of the day: Pride	Recognizing own strengths The emotion of the day: Enthusiasm/Excitement	Recognizing own strengths The emotion of the day: Sadness
Self-regulation I	Becoming familiar with: − Self-regulation − Restless child	The storybook: “Bouncy Bananas Goes Bananas” promotes self-control, negotiating conflict, and overcoming disappointments. The emotion of the day: Restlessness	Learning how to calm down The emotion of the day: Calmness	Identifying calm/calming actions The emotion of the day: Joy
Self-regulation II	Becoming familiar with: − Emotion Regulation − Child’s aggression	The storybook: “Cranky Crabbies’ Hissy Fit” promotes regulating emotions and apologizing. The emotion of the day: Anger/Hate	Learning breathing exercises for calming The emotion of the day: Hurt	Recognizing and expressing emotions The emotion of the day: Relief
Social awareness	Becoming familiar with: − Social awareness − Resilience − Child’s shyness	The storybook: “Feisty Foxy Doesn’t Give Up” promotes helping others, sharing, and perseverance. The emotion of the day: Fear	Learning about resilience and overcoming disappointments The emotion of the day: Satisfaction	Exercises on Pep talk: saying good about others The emotion of the day: Happiness
Relationship skills	Becoming familiar with: − Relationship skills − Bullying − Problem-solving skills	The storybook: “KissKiss Feels Left Out” promotes understanding others’ feelings, kindness, and taking perspectives. The emotion of the day: Feeling left out	Sharing and playing together The emotion of the day: Concern	Working together The emotion of the day: Guilt
Responsible decision making	Becoming familiar with: − Responsible decision making − Envy − Self-esteem − Saying sorry and making up − Confrontation/defiance of the child	The storybook: “Cocoala Wants to be The Best” promotes overcoming envy, admitting own mistakes and apologizing, and giving credit to others.The emotion of the day: Envy	My dreams—drawing exercise The emotion of the day: Astonishment	Relaxing exercises The emotion of the day: Nervousness

**Table 3:** Description of teacher training sessions, workshops’ objectives, skills, and toolkit items.

Workshop 1 Orientation session	Objectives: (1) Enhancing teachers’ knowledge and awareness of SEL and its importance in the ECE system; (2) Introducing the theoretical and pedagogical concepts of the Roundies SEL curriculum, (3) Setting teaching goals for implementing the curriculum. Specific teacher strategies/behaviors: Day-to-day habits to foster a positive teacher-child interaction and build a positive learning environment (e.g., praising each child’s efforts and achievements verbally and nonverbally, greeting each child positively, comforting a distressed child, and praising a child in front of others); Play and playfulness to promote children’s social and emotional skills and decrease their behavior problems. Toolkit items: Emotional cards, Play, Strength cards, Roundies Storybooks, Posters, Wind Map of Emotions, Roadmap of the Program, Coloring exercises, Songs, Roundies breathing exercise cards.
Workshop 2 Self-awareness	Objectives: Enhancing teachers’ knowledge of (1) The development of self-awareness in early childhood; (2) Individual differences and how to use this knowledge to improve teacher-child interactions; (3) Separation anxiety symptoms and how to deal with it; (4) Own self-awareness and acknowledgment of how their own emotions can have an impact on their well-being and children’s SEL. Specific teacher strategies/behaviors: Positive reinforcement and praise to encourage good behaviors; Nonverbal communication (e.g., facial expressions) to help children recognize and understand their own emotions; Group discussions with other teachers to discuss children’s behaviors and temperamental differences; Particular morning routine to help children deal with separation anxiety Toolkit items: Emotional cards, Strength cards, The Roundies storybook, i.e., “Three Cheers for Giraffe”.
Workshop 3 Self-regulation I	Objectives: (1) Enhancing teachers’ knowledge on the development of self-regulation in early childhood; (2) Instructing teachers on how to deal with children’s restlessness and hyperactivity; (3) Increasing teachers’ awareness of the importance of setting daily routines; (4) Participating in a teachers’ discussion group to reflect on the last theme’s goals and setting new goals for the next session. Specific teacher strategies/behaviors: Providing a calm, nurturing, and predictable social and emotional environment in the classroom; Providing children with short, fun, and explicit SEL activities to enhance their concentration and encourage them to calm down; Stating the expectation clearly and providing positive reinforcement; Allocating a particular space in the classroom for performing some routine calming down activities. Toolkit items: Emotional cards and structured discussions, Flamingo’s nest poster, Coloring exercises, The Roundies storybook, i.e., “Bouncy Bananas Goes Bananas”.
Workshop 4 Self-regulation II	Objectives: (1) Deepen teachers’ knowledge of self-regulation in early childhood; (2) Increasing teachers’ knowledge and awareness of children’s aggression behaviors and using the knowledge to improve teacher-child interactions; (3) Familiarizing teachers with some calm-down activities; (4) Enhancing teachers’ own self-regulation skills; (5) Participating in a teachers’ discussion group to reflect on the last theme’s goals and achievements and setting new goals for the next session. Specific teacher strategies/behaviors: Identifying and reducing stressors that trigger outbursts in children; Using active listening to detect the early signs of aggressive behavior; Opening a dialogue with the child about their stress; Teaching children to recognize and manage the feelings and actions that lead to unsafe and aggressive behavior; Using positive verbal feedback; Providing examples of how to say “sorry” and how to handle challenging situations; Using positive reinforcement to draw the child’s attention to good behavior; Teaching children a few quick calming exercises. Toolkit items: A checklist for identifying sources of aggression in the classroom, Visual map of the intensity of feelings, Roundies breathing exercise cards, The Roundies storybook, i.e., “Cranky Crabbies’ Hissy Fit”, Play, Emotional cards, Structured discussions.
Workshop 5 Responsible decision making	Objectives: (1) Increase teachers’ knowledge of responsible decision making in early childhood; (2) Provide some tools for the teachers to set boundaries in positive ways; (3) Enhancing teachers’ awareness of jealousy and envy and how to handle it; (5) Educating teachers on how to foster children’s confidence and self-esteem; (6) Supporting teachers to help children become more responsible; (7) Enhance teachers’ awareness of the importance of apology; (8) Educating teachers on how to deal with confrontation/defiance in children; (9) Participating in a teachers’ discussion group to reflect on the last theme’s goals and setting new goals for the next session. Specific teacher strategies/behaviors: Treating children equally to prevent envy; Giving positive feedback based on each child’s strengths; Verbalizing the situations that create jealousy to calm the situation; Teaching children to celebrate their individuality; Engaging children when making rules; Telling children clearly what is expected of them; Giving the children time to choose to follow the rule; Using nonverbal ways to set boundaries; Showing affection for the child; Providing children with appropriate tasks; Praising the children when trying and when achieving; Creating a list of how to make up after a conflict; Try not to make it a game of power and get carried away in “yes but, no but”-conversations; Try to distract the child; Observe the child’s needs; Let the child rehearse decision making by choosing one of two alternatives. Toolkit items: Emotional cards and structured discussions, Visual Making up –list for the children to understand ways of giving an apology, The Roundies storybook, i.e., “Cocoala Wants to be The Best”.
Workshop 6 Social awareness	Objectives: (1) Enhance teachers’ knowledge of the development of social awareness in early childhood; (2) Supporting teachers in teaching children how to recognize and understand others’ perspectives and feelings, empathize with them, and offer help when needed; (3) Supporting teachers’ understanding of shyness in early childhood; (3) Supporting teachers to foster resilience in ECE settings; (4) Participating in a teachers’ discussion group to reflect on the last theme’s goals and setting new goals for the next session. Specific teacher strategies/behaviors: Modeling how to show compassion and empathy towards others; Creating opportunities to practice taking another’s perspective; Encouraging children to offer kind and supportive words to a stressed child; Setting clear expectations; Encouraging children to think and talk about how the other person might be feeling in a particular situation; Practice active listening to each other’s perspectives; Supporting children’s resilience; Motivating children to engage in SEL by engaging them in activities that they are naturally interested in; Teaching children when to try their best and when to give up; Supporting shy children; Acknowledging other positive attributes of children instead of referring them as being shy. Toolkit items: Emotional cards and structured discussions, Play; Writing individual “praise posts”; The Roundies storybook, i.e., “Feisty Foxy Doesn’t Give Up”.
Workshop 7 Relationship skills	Objectives: (1) Enhancing teachers’ knowledge of the development of relationship skills in early childhood; (2) Supporting teachers in how to improve children’s social skills and peer relationships; (3) Increase the awareness and understanding of bullying; (4) Enhancing teacher’s knowledge of how to develop children’s problem-solving skills; (5) Participating in a teachers’ discussion group to reflect on the last theme’s goals and setting new goals for the next session. Specific teacher strategies/behaviors: Creating a “friendship bench,” i.e., a place for children to sit when they feel lonely or in need of a friend to play with so that others could notice them and take them along; Creating opportunities to practice practical social skills and encouraging children to connect with peers; Facilitating collaborative learning in the classroom; Giving children an active role in solving conflicts; Calming the child down before solving the conflict. Toolkit items: Emotional cards and structured discussions; Play; The Roundies storybook, i.e., “KissKiss Feels Left Out”.
Workshop 8 End session	The last session for teachers included concluding all the SEL program’s theoretical concepts and evaluating its impact. The teachers were asked to discuss: (1) Whether the program’s objectives were met; (2) Whether the children’s social-emotional skills enhanced; (3) Whether children’s problem behaviors decreased; (4) Whether the teachers’ own well-being improved. The teachers’ final session also included planning some fun exercises for the last children’s session, i.e., “End celebration”.

**Table 4:** Teacher perception of the Roundies program effectiveness and satisfaction with the program components.

Satisfaction with the Program in General *	
The program made some changes that improved the way things work in my classroom.	100%
I feel the program had a positive impact on my relationship with children.	95%
I have seen positive changes in children’s behaviors.	95%
I can see improvement in children’s emotional expressions in the classroom.	95%
I see myself using the program exercises in daily classroom activities after the intervention.	100%
I would recommend the Roundies program to other ECE teachers.	100%
Satisfaction with the program components **	
Online learning materials	87%
Teachers’ discussion groups	91%
The handbook	87%
Toolkit items (e.g., the Roundies storybooks, emotional cards, relaxing exercises, etc.)	95%

Note. * Percent of teachers who indicated that they “agreed’’ or ‘‘strongly agreed”; ** Percent of teachers who viewed each program component as helpful.

**Table 5:** Multilevel models explaining the prosocial post-test score (*N* = 194).

Model	1	2	3	4
Intercept	4.644 (0.158) ****	4.684 (0.133) ****	4.201 (0.222) ****	4.200 (0.223) ****
Fixed Effects				
Pre-test score		0.717 (0.078) ****	0.733 (0.077) ****	0.762 (0.156) ****
Gender (girls as reference)		−0.312 (0.129) **	−0.297 (0.128) **	−0.295 (0.129) **
Age		0.016 (0.007) **	0.015 (0.007) **	0.015 (0.007) **
Condition (control group as reference)			0.650 (0.285) **	0.650 (0.259) **
Pre-test score * Condition				−0.037 (0.176)
Random Effects				
Variance of the within-level residual errors	0.725 (0.078) ****	0.408 (0.057) ****	0.409 (0.058) ****	0.409 (0.058) ****
Variance of the between-level residual errors	0.478 (0.166) ***	0.222 (0.100) **	0.139 (0.072) *	0.140 (0.073) *
Deviance (df)	530.036 (3)	252.336 (6)	247.066 (7)	247.022 (8)
Decrease in Deviance		277.7 ****	6 **	0.044

Note. **** = *p* < 0.0001, *** = *p* < 0.001, ** = *p* < 0.01, * = *p* < 0.05.

**Table 6:** Multilevel models explaining the SDQ total difficulties post-test score (*N* = 194).

Model	1	2	3	4
Intercept	2.801 (0.128) ****	2.796 (0.072) ****	3.050 (0.122) ****	2.879 (0.072) ****
Fixed Effects				
Pre-test score		0.816 (0.052) ****	0.823 (0.051) ****	0.835 (0.060) ****
Gender (girls as reference)		0.110 (0.084)	0.099 (0.083)	0.103 (0.084)
Age		−0.013 (0.004) ***	−0.012 (0.004) ***	−0.013 (0.004) ***
Condition (control group as reference)			−0.338(0.142) **	−0.333 (0.145) **
Pre-test score * Condition				−0.048 (0.123)
Random Effects				
Variance of the within-level residual errors	0.492 (0.053) ****	0.177 (0.025) ****	0.177 (0.025) ****	0.177 (0.025) ****
Variance of the between-level residual errors	0.308 (0.111) ***	0.057 (0.033) *	0.035 (0.024)	0.032 (0.025)
Deviance (df)	454.966 (3)	148.456 (6)	143.496 (7)	143.354 (8)
Decrease in Deviance		306.51 ****	4.96 **	0.04

Note. **** = *p* < 0.0001, *** = *p* < 0.001, ** = *p* < 0.01, * = *p* < 0.05.

## Data Availability

The data presented in this study are available on request from the corresponding author. The data are not publicly available due to privacy and ethical considerations.

## Supplementary Materials

The following is available online at https://www.mdpi.com/article/10.3390/ijerph182010679/s1, File S1: The description of teacher training sessions.
