# Supplementary material for: Training Early Childhood Teachers to Support Children’s Social and Emotional Learning: A Preliminary Evaluation of Roundies Program
Source: Int J Environ Res Public Health. 2021 Oct 12;18(20):10679. doi: 10.3390/ijerph182010679 (PMC8535282; doi:10.3390/ijerph182010679)
Supplement: Supplementary file 1 [file ijerph-18-10679-s001.zip › ijerph-1355346-supplementary.pdf]

Online appendix for - Training Early Childhood Teachers to Support Children's Social and Emotional Learning: A Preliminary Evaluation of Roundies Program by Moazami-Goodarzi et al.

Supplementary Material: The description of teacher training sessions

The detailed descriptions of the eight teacher training sessions that address the five components of SEL are as follows:

Workshop 1 – Orientation session

Objectives: 1) Enhancing teachers' knowledge and awareness of SEL and its importance in the ECE system; 2) Introducing the theoretical and pedagogical concepts of the Roundies SEL curriculum, particularly the framework for fostering a positive learning environment and its implementation; 3) Setting some teaching goals for implementing the curriculum during the intervention program.

Specific teacher strategies/behaviors: Day-to-day habits to foster a positive teacher-child interaction and build a positive learning environment, such as praising each child's efforts and achievements verbally and nonverbally, greeting each child positively, comforting a distressed child, and praising a child in front of others; Play and playfulness to promote children's social and emotional skills and decrease their behavior problems.

Toolkit items designed to help teacher practice: "Emotional cards" to help children to recognize and name their own feelings and emotions; "Play" to support the children's natural way of learning (constructive play, social play, physical play, ball games, drama exercises, mime exercise etc.); "Strength cards," to help children to be aware of and recognize different strengths and to find the strengths in themselves and others; "Roundies Storybooks" to build children's SEL skills; "Posters" to help visualize processes and symbols for children (such as "Flamingo nest" to mark a place for calming down, or the "Wind Map of Emotions" to help children verbalize and visualize the intensity of their emotions, "Roadmap of the Program" to help the children understand the entirety of the program); "Coloring exercises" such as "Strength Totem" to draw attention to the theme and provide something calming to do while discussing about the theme; "Songs" to engage children with the program themes; "Roundies breathing exercise cards" to help children calm down physically and mentally.

Workshop 2 – Self-awareness

Objectives: 1) Enhancing teachers' knowledge of the development of self-awareness in early childhood and how to lead children through the process of understanding what they experience; 2) Educating teachers on individual differences and how to use this knowledge to improve teacher-child interactions; 3) Enhancing teachers' awareness of separation anxiety symptoms and how to deal with it; 4) Enhancing teachers' own self-awareness and acknowledgment of how their own emotions can have an impact on their well-being and children's SEL.

Specific teacher strategies/behaviors: Positive reinforcement and praise to encourage good behaviors; Nonverbal communication, e.g., facial expressions and verbal communication, to help children recognize and understand their own emotions; Group discussions with other teachers to discuss children's behaviors and temperamental differences; Particular morning routine to help children deal with separation anxiety, e.g., telling a lovely story about the morning or presenting an object such as a picture to remind the child of their parent).

Toolkit items designed to help teacher practice: Emotional cards to help children recognize and understand pride, enthusiasm/excitement, and sadness; Strength cards to help children identify their own strengths; The Roundies storybook, i.e., "Three Cheers for Giraffe" to encourage children to overcome their fears, recognize their emotions and strengths, and talk about their feelings with other children.

Workshop 3 – Self-regulation I

Objectives: 1) Enhancing teachers' knowledge on the development of self-regulation in early childhood and how to help children regulate their own emotions, thoughts, and behaviors; 2) instructing teachers on how to deal with children's restlessness and hyperactivity; 3) Increasing teachers' awareness of the importance of setting daily routines to provide a framework and guide for behavioral expectations and procedures throughout the day; 4) Participating in a teachers' discussion group to reflect on the last theme's goals and achievements and setting new goals for the next session.

Specific teacher strategies/behaviors: Providing a calm, nurturing, and predictable social and emotional environment to help children feel comfortable and control their choices and reduce their anxiety and restlessness; Providing children with short, fun, and explicit SEL activities enhances their concentration and encourage them to calm down; Stating the expectation clearly verbally and nonverbally and providing positive reinforcement to help children manage their behaviors and emotions; Allocating a particular space in the classroom for performing some routine calming down activities.

Toolkit items designed to help teacher practice: Emotional cards and structured discussions to help children recognize and understand restlessness, calmness, and joy; Flamingo's nest –poster to create a physical space for the children to calm down; Using coloring tools to help children calm down; The Roundies storybook, i.e., "Bouncy Bananas Goes Bananas" which promotes self-control, negotiating conflict, and overcoming disappointments.

Workshop 4 – Self-regulation II

Objectives: 1) Deepen teachers' knowledge of self-regulation in early childhood, particularly how to support children in improving their emotion-regulation skills; 2) Increasing teachers' knowledge and awareness of children's aggression behaviors and using the knowledge to improve teacher-child interactions and eliminate the feelings of loss of control, helplessness, and shame in children; 3) Familiarizing teachers with some calm-down activities to enhance children's relaxation coping skills; 4) Enhancing teachers' own self-regulation skills by reflecting on the ways that they regulate emotions and release stress, and what kind of support they can get from each other in challenging situations; 5) Participating in a teachers' discussion group to reflect on the last theme's goals and achievements and setting new goals for the next session.

Specific teacher strategies/behaviors: Identifying and reducing stressors that trigger outbursts in children to eliminate aggressive behavior; Using active listening to detect the early signs of aggressive behavior; opening a dialogue with the child about their stress; Teaching children to recognize and manage the feelings and actions that lead to unsafe and aggressive behavior; Using positive verbal feedback to support the child's capacity to regulate their emotions; Providing examples of how to say "sorry" and how to handle challenging situations; Using positive reinforcement to draw the child's attention to good behavior; Teaching children a few quick calming exercises such as taking a deep breath.

Toolkit items designed to help teacher practice: A checklist for identifying sources of aggression in the classroom; Visual map of the intensity of feelings, i.e., "Wind map of emotions," to help children in recognizing the intensity of their emotions (e.g., calm weather, breeze, storm, or hurricane level of intensity); Roundies breathing exercise cards to help children calm down physically and mentally; The Roundies storybook, i.e., "Cranky Crabbies' Hissy Fit" which promotes emotion regulation and apologizing; Play and discussion to help children to recognize and express different emotions and their intensity; Emotional cards and structured discussions to help children identify and understand anger, hate, hurt and relief.

Workshop 5 – Responsible decision-making

Objectives: 1) Increase teachers' knowledge of responsible decision-making in early childhood; 2) Provide some tools for the teachers to set boundaries in positive ways; 3) Enhancing teachers' awareness of jealousy and envy among children and how it can affect the group; 4) Supporting teachers in teaching children how to handle envy and how to overcome it; 5) Educating teachers on how to foster children's confidence and self-esteem; 6) Supporting teachers to help children become more responsible; 7) Enhance teachers' awareness of the importance of apology and how they can teach children to apologize; 8) Educating teachers on how to deal with confrontation/defiance in children; 9) Participating in a teachers' discussion group to reflect on the last theme's goals and achievements and setting new goals for the next session.

Specific teacher strategies/behaviors: Treating children equally and not favoring one child over another to prevent envy; Giving positive feedback based on each child's strengths and showing the child that you like to spend time with him/her to support self-esteem and to avoid jealousy; Verbalizing the situations that create jealousy to calm the situation; Teaching children to celebrate their individuality, create a workable plan, and guide them in achieving the goal; Engaging children when making rules to encourage them to follow the rules and differentiate right from wrong; Telling children clearly what is expected of them; Giving the children time to choose to follow the rule before repeating it; Using nonverbal ways to set boundaries, e.g., making eye-contact with the child and gently moving him/her away from the unwanted action, distracting the child's attention from unwanted activity towards something interesting, not commenting on every little wrong action and instead giving positive comments when the child follows the rule; Showing affection for the child by active listening, giving him/her a hug, comforting the child to let the child feel she/he is safe and loved; Providing children with appropriate tasks to challenge them a little but also allow them to succeed; Praising the children when trying and when achieving; Creating a list of how to make up after a conflict and how to practice apologizing and being responsible; To cope with confronting a child, prepare the

child beforehand for the transitions; Try not to make it a game of power and get carried away in "yes but, no but"-conversations; Try to distract the child; Observe the child's needs and see how you could respond to the demands; Let the child rehearse decision making by choosing one of two alternatives.

Toolkit items designed to help teacher practice: Emotional cards and structured discussions to help children recognize and understand envy, astonishment, and nervousness; Visual Making up –list for the children to understand ways of giving an apology; The Roundies storybook, i.e., "Cocoala Wants to be The Best" which promotes overcoming envy, accepting own mistakes, apologizing, and giving credit to others.

Workshop 6 – Social awareness

Objectives: 1) Enhance teachers' knowledge of the development of social awareness in early childhood; 2) Supporting teachers in teaching children how to recognize and understand others' perspectives and feelings, empathize with them, and offer help when needed; 3) Supporting teachers' understanding of shyness in early childhood, how to detect the signs, and how to deal with it; 3) Supporting teachers to foster resilience in ECE settings; 4) Participating in a teachers' discussion group to reflect on the last theme's goals and achievements and setting new goals for the next session.

Specific teacher strategies/behaviors: Modeling how to show compassion and empathy towards others; Creating opportunities to practice taking another's perspective and imagining what others are thinking; Encouraging children to offer kind and supportive words to a stressed child; Setting clear expectations letting children know that they need to care about one another; Encouraging children to think and talk about how the other person might be feeling in a particular situation such as when a conflict arises; Practice active listening to each other's perspectives; Supporting children's resilience by acknowledging even their most minor attempts and efforts and giving them positive feedback; Motivating children to engage in SEL by engaging them in activities that they are naturally interested in; Teaching children when to try their best and when to give up; Supporting shy children by encouraging them, not forcing them, and give them more time to adjust; Supporting shy children to feel comfortable in the situations and by treating them like others although avoiding putting them suddenly at the center of attention; Acknowledging other positive attributes of children instead of referring them as being shy.

Toolkit items designed to help teacher practice: Emotional cards and structured discussions to help children recognize and understand fear, satisfaction, and happiness; Play to help children understand it's OK to make mistakes and that they can always try again; Writing individual "praise posts" and handing them to others to help children acknowledge others' feelings and needs and express empathic concern for others; The Roundies storybook, i.e., "Feisty Foxy Doesn't Give Up" which promotes helping others, sharing, and perseverance.

Workshop 7 – Relationship skills

Objectives: 1) Enhancing teachers' knowledge of the development of relationship skills in early childhood; 2) Supporting teachers in how to improve children's social skills and relationships with peers; 3) Increase the awareness and understanding of bullying and supporting teachers in preventing bullying and promoting constructive communication; 4) Enhancing teacher's knowledge of how to develop children's problem-solving skills;

5) Participating in a teachers' discussion group to reflect on the last theme's goals and achievements and setting new goals for the next session.

Specific teacher strategies/behaviors: Creating a "friendship bench," i.e., a place for children to sit when they feel lonely or in need of a friend to play with so that others could notice them and take them along; Creating opportunities to practice practical social skills and encouraging children to connect with peers; Facilitating collaborative learning in the classroom; Giving children an active role in solving conflicts to develop their problem-solving skills; Calming the child down before solving the conflict.

Toolkit items designed to help teacher practice: Emotional cards and structured discussions to help children recognize and understand the feeling of being left out, concern, and guilt; Play to help build a sense of community in the classroom, teach children how to share and cooperate with others; The Roundies storybook, i.e., "KissKiss Feels Left Out" which promotes understanding others' feelings, kindness, and taking perspective.

Workshop 8 – End session.

The last session for teachers included concluding all the SEL program's theoretical concepts and evaluating its impact. The teachers were asked to discuss: 1) Whether the program's objectives were met; 2) Whether the children's social-emotional skills enhanced; 3) Whether children's problem behaviors decreased; 4) Whether the teachers' own well-being improved. The teachers' final session also included planning some fun exercises for the last children's session, i.e., "End celebration".
